# Toll-Like Receptors Signaling Contributes to Immunopathogenesis of HBV Infection

**DOI:** 10.1155/2011/810939

**Published:** 2011-11-29

**Authors:** Yasuteru Kondo, Yoshiyuki Ueno, Tooru Shimosegawa

**Affiliations:** Division of Gastroenterology, Tohoku University Hospital, 1-1 Seiryo-Machi, Aoba-ku, Miyagi, Sendai City 980-8574, Japan

## Abstract

Innate and adaptive immune systems have important role in the pathogenesis of acute and chronic infection with hepatitis B virus (HBV). These immune responses are mediated through complex interactions between the innate immune response and adaptive immune response. Toll-like receptors (TLRs) are a family of innate immune-recognition receptors that recognize the molecular patterns associated with microbial pathogens. So far, TLR1 to 13 were found in human or mice and investigated to detect the target molecules and the downstream mechanisms of these unique systems. Stimulation by their ligands initiates the activation of complex networks of intracellular signaling transduction and innate and adaptive immune-related cells (NK, NK-T, monocytes, dendritic cells, T cells, B cells, and Tregs, etc.). However, reports on such relationships between HBV and TLRs have been relatively rare in comparison to those on HCV and TLRs, but have recently been increasing. Thus, a review of TLRs involved in the pathogenesis of HBV infection may be needed toward better understanding of the immunopathogenesis of HBV infection.

## 1. Introduction

HBV is an enveloped, double-strand DNA virus that causes acute, chronic hepatitis and hepatocellular carcinoma (HCC) [1]. HBV infection continues to be a significant global health problem. There are approximately 400 million chronic HBV carriers in the world, and in approximately 5% of adults and 95% of neonates who become infected with HBV, persistent infection develops [2]. 

It has been shown that the innate immune system including natural killer (NK) cells, NK-T cells, monocytes, and intrahepatocyte-immune reaction in addition to the adaptive immune system including cytotoxic T lymphocyte (CTLs), Th1 CD4+ cells, CD4+CD25+FOXP3+ regulatory T cells (Tregs), and dendritic cells (DCs) plays an important role in the control of virus [3–8]. The hyporesponsiveness of NK cells, HBV-specific Th1 cells and distortion in regulatory functions of Tregs have been demonstrated in CHB patients [9, 10]. Lamivudine treatment in CHB has been reported to restore both CD4+ and CTL hyporesponsiveness following the decline of serum levels of HBV-derived Ag by many groups including us [11]. However, the other group reported that this restoration of T-cell response might be transient [12]. We could not conclude whether the reduction of hepatitis B viral antigens could contribute to the better response of immune therapy. We previously reported that HBcAg-specific IL10 secreting Tregs could have important roles in the pathogenesis of CHB [13]. More recently, we found that one of the stress-related proteins produced by HBV-replicating hepatocytes could enhance the suppressive activity of Tregs via TLR2 [14]. In addition, HBV antigens and proteins related to HBV replication were reported to contribute to the TLR-related immune reactions [15]. 

TLRs are the most common groups among the pattern recognition receptors (PRRs), which differ from antigen-specific receptors in a number of ways. The TLRs were initially identified based on homology with the toll receptor from Drosophila. They are expressed on many kinds of cells including hepatocytes and effector cells of the innate immune and adaptive immune systems. Thus, TLRs might contribute not only to the innate immune systems but also to the adaptive immune systems. TLRs can be classified into two groups based on subcellular localization. The first group includes TLR1, 2, 4, 5, 6, 11, and 12, which are present at plasma membrane (Table 1). The second group includes TLR3, 7, 8, and 9, which localize to intracellular compartments such as endosomes [16]. Once the TLRs recognize a pathogen-associated molecular pattern, the effector cell is triggered to perform its function immediately, rather than after proliferation in the lymph nodes. Stimulation is started through the involvement of adaptor protein MyD88, protein kinase, and the transcription factor, nuclear factor kappa B (NF-*κ*B), which can induce a variety of cytokines including TNF-*α*, IL-1, and IL-6. Thereafter, stimulation of TLRs could contribute to the coordination of inflammatory immune responses. So far, TLR2, 3, 4, 7, 8, and 9 might have roles in the pathogenesis of various kinds of viral infections including hepatitis B and C (Table 1).

## 2. TLRs and HBV Replicating Hepatocyte

**Figure 1:**
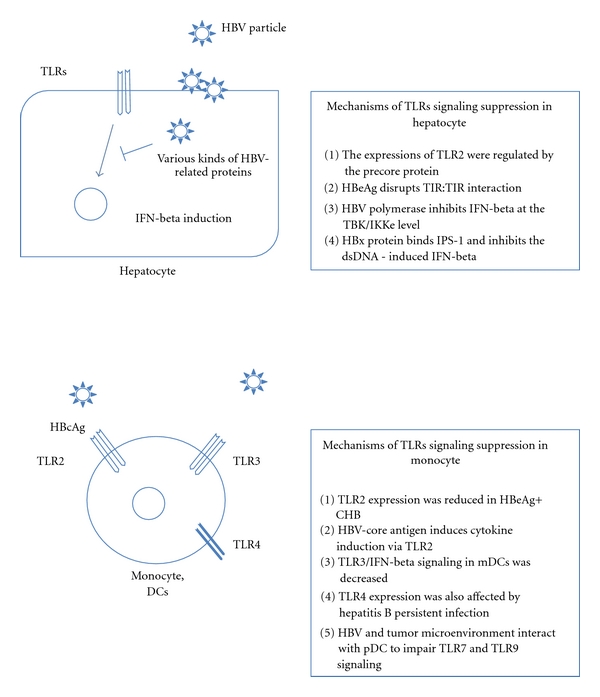
TLRs contribution to Hepatocellular- and monocyte-related immune systems.

The initial step of the innate immune response is induced by type 1 IFN secretion from infected hepatocytes via stimulation of TLRs in hepatitis C virus infection [17, 18]. However, the relationship between HBV and TLRs in hepatocyte has been relatively unclear in comparison to that between HCV and TLRs. It has been reported that the expression of TLR2 is regulated by the precore protein in CHB, and that there was significantly reduced cytokine production (TNF-alfa) and phospho-p38 kinase expression in the presence of HBeAg [19]. More recently, the same group reported that HBeAg colocalized with the Toll/IL-1-receptor-(TIR-) containing proteins TRAM, MAL, and TLR at the subcellular level. Thus, HBeAg was able to disrupt TIR:TIR interaction critical for TLR-mediated signaling [20]. Another group reported that HBV polymerase might inhibit IFN-beta expression at the TBK/IKKe level. IFN-beta induction is one of the first-phase characteristics of the activation of the type I IFN system [21]. In addition to these reports, other groups have described that HBV could suppress TLRs signaling in hepatocytes as well as nonparenchymal liver cells [22]. These reports were very important, since direct disruption of TLRs signaling was demonstrated in CHB patients as seen in CHC patients (Figure 1). For example, HCV NS3/4A could cleave TRIF and IPS-1 and lead to a deficiency of IFN-beta induction in HCV-infected hepatocytes [18]. Not only TLRs but also retinoic acid-inducible gene I (RIG-I) might be involved in the pathogenesis of HBV infection. Kumar et al. reported that HBx protein (the 17 kDa HBV regulatory protein that plays an essential role in virus replication) could bind to adaptor protein IPS-1 and inhibit the dsDNA-induced IFN-beta [23]. 

## 3. TLRs Expression on Monocytes and DCs in HBV Infection

**Figure 2:**
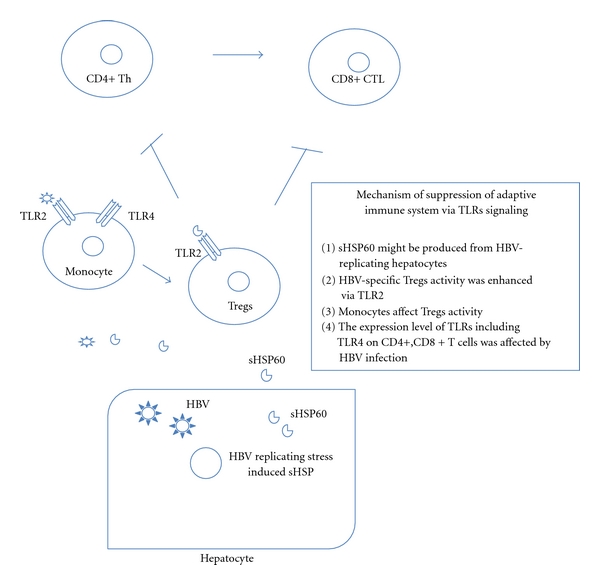
TLRs contribution to PBMC, CD3+/CD4+/CD8+ T cells, and Tregs-related immune systems.

Monocytes including Kupffer cells play an important role in mediating the innate and adaptive immune responses. Various kinds of TLRs are highly expressed on monocytes in comparison to NK cells and CD3+ lymphocytes. It was reported that TLR2 expressions on peripheral monocytes were reduced in patients with HBeAg+ CHB [24]. In addition to the TLR2 expression, another group clearly demonstrated that the cytokine induction by the HBV capsid in macrophages is facilitated by the interaction of its arginine-rich domain with membrane heparan sulfate and involves signaling through TLR2. They found the mechanisms of cytokine induction by HBV core Ag (HBcAg), which serves as the structural subunit of the highly immunogenic capsid shell. HBcAg harbors a unique arginine-rich C terminus that has been implicated in the immune responses induced by the capsid [15]. Although it has also been reported that no quantitative, phenotypic of functional impairment of mDCs or pDCs was found in CHB [25], TLR3/IFN-beta signaling was decreased significantly in mDCs from patients with chronic hepatitis B or acute-on-chronic hepatitis B liver failure, compared with normal controls [26] (Figure 1). Moreover, the existence of a relationship between TLR2/4 expressions on monotyes and Tregs in CHB patients was reported recently [27] (Figure 2). In addition to TLR2/4, impairment of TLR7 and TLR9 signaling might be associated with chronic viral infection including HIV-1, HBV, and HCV. One group reported that viruses and components of the tumor microenvironment interact with regulatory receptors on plasmacytoid dendritic cells to impair TLR7 and TLR9 signaling.

## 4. TLRs Expression on PBMC, CD3+/CD4+/CD8+ T Cells, and Tregs in HBV Infection

For the resolution of viral infection, the efficient recognition of intracellular viruses is essential. It has been reported that CTLs and Th1 play a central role in the control of viral infection after the rapid response by NK and NK-T cells [4]. CTL recognizes viral antigens synthesized within infected cells in the form of oligopeptides that are presented on HLA class I molecules. Many groups including us have reported on the hyporesponsiveness of HBV-specific CTLs in the peripheral blood of patients with CHB [11, 28–30]. There may be several mechanisms in this CTLs hyporesponsiveness including anergy, exhaustion induced by the large amount of HBV antigens, PD-1: PD-L1 interaction, and excessive function of Tregs during CHB [3, 11, 13, 14, 31–36]. Among these immune cells that could contribute to the cellular immune responses, the involvement of Tregs was investigated intensely [13, 14, 27, 34–41]. However, although the expression levels of TLRs were relatively low in comparison to monocytes and DCs, the biological significance of TLRs expression on a variety of T cells was substantial [27, 34, 36–44]. It has been shown that TLR2 and 4 were important for the regulation of Tregs [42–44]. More recently, we have shown that the soluble form of heat shock protein 60 induced by HBV-replicating hepatocytes could enhance the HBcAg-specific Il10 secreting activity of Tregs via TLR2 (Figure 2). The suppression of HBV replication with entecavir therapy could reduce the excessive function of Tregs in CHB [14]. Based on other findings and those of our studies, not only the HBV antigens but also unknown proteins produced from HBV-replicating hepatocytes and other cells could affect the immune function via TLRs.

## 5. Immunological Therapy with TLR Agonist

Interferon and nucleos(t)ide analogs, such as lamivudine, adefovir, entecavir, and tenofovir, are the currently available treatments. Interferon-alfa is effective in approximately 30% of cases and even less so in younger cases [45]. The efficacy of nucleos(t)ide analogs is limited by viral reactivation because of the emergence of escaped mutants in cases of prolonged treatment [46, 47]. Immunotherapy is one of the important options to control HBV replication. Various kinds of immune therapies including protein vaccines, DNA vaccine, T-cell epitope vaccines, and immunomodulating agents (cytokines, IFN, and TLR agonists) have been reported [48–51].

So far, a promising immune therapy that could eradicate or control HBV has not been developed. Cellular immune responses are suppressed by various kinds of mechanisms. Therefore, TLR agonists were strong candidates to activate and modulate the impaired immune responses with HBV epitopes, proteins, and DNA vaccines [52–57]. Whether TLR2, 3, 4, 5, 7, and 9 signaling could enhance the CTL response upon vaccination has been examined. The results indicated that TLR2 and 4 failed to increase the CTL response, whereas stimulation by TLR3, 5, and 7 exhibited a moderate adjuvant function. In contrast, stimulation of TLR9 dramatically increased the CTL responses [54]. On the other hand, TLR4 in addition to TLR 3, 5, 7, and 9 was reported to have the ability to suppress HBV replication in a mouse model [52, 53]. Thereafter, a vaccine based on HBV antigens and immunostimulatory DNA sequence, an adjuvant that acts as a TLR9 agonist, was developed [55]. However, to determine the potential of using TLR agonists to improve the HBV-specific immune response, future studies will be needed. 

## 6. Concluding Remarks

Recently various studies about the immunopathogenesis of HBV infection, including adaptive and innate immune responses, have been reported, and the contribution of TLRs signaling to the pathogenesis of HBV infection is receiving increased interest. In this paper, we described the suppression of TLRs signaling in heptocytes and immune cells during HBV infection, however, reports concerning the suppression of TLRs signaling in HBV infection have been few in comparison to those on HCV infection. The involvement of TLRs signaling suppression during HBV persistent infection should be considered more precisely. For example, the relationship between HBV genotypes and the level of TLRs suppression is not clear yet. As for immune therapy, the TLRs agonists, especially TLR9 agonist, with various kinds of HBV vaccines could be strong candidates to activate the HBV-specific immune responses that are suppressed during HBV persistent infection. The combination of TLR agonists and appropriately timed immune therapy should be considered in future studies.

## Figures and Tables

**Table 1:** TLR signaling affecting the immunopathogenesis of HBV infection.

TLRs	Localization	Ligand/Pathogen-associated molecular pattern	Contribution to the pathogenesis of HBV infection
TLR1	Plasma membrane	Triacyl lipopeptides, soluble factors	Ligand of TLR1 might suppress HBV replication
TLR2	Plasma membrane	Lipoprotein/lipopeptide	TLR2 expressions on monocytes and hepatocytes were reduced in HBeAg+ CHB sHSP60 produced from HBV-replicating hepatocyte enhanced Tregs activity via TLR2 HBeAg suppresses activation of the TLR2 signaling
TLR3	Endosomes	Double-strand RNA	HBV polymerase inhibits RIG-I- and TLR3-mediated beta interferon induction Ligand of TLR3 might suppress HBV replication Moderate adjuvant function
TLR4	Plasma membrane	LPS, fusion protein	Polymorphisms of TLR4 gene may be related to protection from HBV recurrence after liver transplantation Ligand of TLR4 might suppress HBV replication via CTL activation
TLR5	Plasma membrane	Flagellin	Moderate adjuvant function
TLR6	Plasma membrane	Diacyl lipopeptides	
TLR7	Endosomes	single-strand RNA	Moderate adjuvant function
TLR8	Endosomes	Imidazoquinolne, single-strand RNA	
TLR9	Endosomes	CpG DNA	Strongly enhances CTL activation; HBV vaccine with TLR9 ligand might be effective
TLR11	Plasma membrane	Profilins-like protein	
TLR12	Plasma membrane	not clear	
TLR13		Vesicular stomatitis virus	
